# Kinetics of Viral Genome Distribution in Swine Peripheral Lymphoid Organs Following Oronasal Infection with Attenuated African swine fever virus strains

**DOI:** 10.3390/v17111472

**Published:** 2025-11-04

**Authors:** Kalhari Goonewardene, Carissa Embury-Hyatt, Estella Moffat, Aruna Ambagala

**Affiliations:** 1 National Centre for Foreign Animal Disease, Canadian Food Inspection Agency, Winnipeg, MB R3E 3M4, Canada; kalhari.goonewardene@inspection.gc.ca (K.G.); carissa.embury-hyatt@inspection.gc.ca (C.E.-H.); estella.moffat@inspection.gc.ca (E.M.); 2 Department of Veterinary Pathology, Western College of Veterinary Medicine, University of Saskatchewan, Saskatoon, SK S7N 5B4, Canada; 3 Department of Medical Microbiology and Infectious Diseases, Max Rady College of Medicine, University of Manitoba, Winnipeg, MB R3E 0J9, Canada; 4 Faculty of Veterinary Medicine, University of Calgary, Calgary, AB T2N 1N4, Canada

**Keywords:** ASF, distribution, peripheral lymph nodes, kinetics, histopathology, immunohistochemistry

## Abstract

African swine fever (ASF) continues to spread across the globe, causing a severe impact on the swine industry. Passive surveillance based on testing dead pigs is one of the most effective methods for early detection of ASF incursions. We have previously shown that the superficial inguinal lymph node (SILN) is a convenient and effective sample type for ASF virus (ASFV) genome detection in pigs succumbed to highly or moderately virulent ASFV infections. In this study, we explored the distribution kinetics of ASFV into SILN and other lymphoid tissues in pigs exposed to moderately virulent ASFV strains (ASFV Estonia 2014 and ASFV Malta’78), oronasally. The ASFV genome was detected in SILNs as early as 2–3 days post-infection (dpi), peaking around 5–9 dpi. The detection of ASFV Estonia 2014 started early, and the pigs succumbed to infection faster compared to the ASFV Malta’78 infected pigs that remained longer. All pigs that succumbed to ASF had comparable levels of ASFV genomic material in the spleen and SILNs. The levels of ASFV genomic material gradually started to decrease in pigs that did not succumb to ASF, indicating possible virus clearance. In contrast, ASFV genome levels in blood and spleen samples remained relatively steady during the study period. Immunohistochemistry and in situ hybridization of spleen and SILN samples supported real-time PCR results. This study demonstrates the distribution kinetics of moderately virulent ASFV in peripheral lymph nodes and highlights the utility of SILNs for dead pig screening.

## 1. Introduction

African swine fever (ASF), a fatal hemorrhagic disease in swine, continues to cause outbreaks globally with serious implications for worldwide swine health, trade, and the global economy. Since the emergence of ASF in Georgia in 2007 [1,2], it has spread to Russia [3], Eastern and Western Europe [4], Asia, and into the island of Hispaniola [5]. ASF virus (ASFV) is a large complex, enveloped DNA virus with an icosahedral structure belonging to the genus *Asfivirus* within the *Asfarviridae* virus family. The clinical presentation of ASF can vary depending on the virulence of the virus responsible. Highly virulent ASFV strains are responsible for per-acute and acute forms of ASF characterized by high fever, lack of appetite, lethargy, hemorrhages, and 100% mortality [6,7,8]. Pigs infected with moderately virulent strains show milder clinical signs, and some pigs may survive the infection [6,9]. Low-virulent strains cause subclinical non-hemorrhagic infections characterized by necrotic skin lesions and joint swellings [6,9,10,11]. Due to the variable and non-specific nature of the clinical signs, it is imperative to perform laboratory diagnostics to confirm ASF.

Passive surveillance based on testing dead pigs is one of the most effective methods for the early detection of ASF incursions [12,13,14,15]. World Animal Health Organization (WOAH) recommends the collection and testing of tissue samples, such as spleen, lymph nodes, bone marrow, lung, tonsil, and kidney from dead or euthanized animals, when there is a suspicion of ASF [16]. However, harvesting the WOAH recommended sample types poses significant challenges since they demand invasive sampling techniques that require opening the carcasses, which is time-consuming and requires additional skilled labor [17] and often leading to contamination of the premises [18]. Due to these reasons, exploring alternative easily accessible sample types/matrices, such as superficial lymph nodes, is important for passive surveillance efforts [19]. We have previously shown that superficial inguinal lymph nodes (SILN) can be easily collected and used as a convenient and effective sample type for ASFV genome detection in pigs that died of highly or moderately virulent ASFV [20]. In that study, we demonstrated that ASFV genome copy numbers in SILNs were highly correlated with those in the spleen, and by sampling SILNs, 100% of the pigs that died of ASF can be positively identified. The objective of the present study was to evaluate the kinetics of ASFV distribution in SILNs and other lymphoid tissues in pigs experimentally infected with two moderately virulent ASFV strains, ASFV Estonia 2014 (p72 genotype II) and ASFV Malta 78’ (p72 genotype I) by natural oronasal route, and to determine if SILN can also be used to detect ASFV in pigs showing early clinical signs, and those that survive the infection.

## 2. Materials and Methods

### 2.1. Animals

In this study, we analyzed archived whole blood, spleen, and superficial lymph nodes, including SILN, submandibular lymph nodes (SMLN), superficial cervical lymph nodes (SCLN), popliteal lymph nodes (PLN), pre-femoral lymph nodes (PFLN), and gastro hepatic lymph nodes (GHLN). The samples were collected from two independent animal experiments (39–40 pigs in each) conducted at the National Center for Foreign Animal Disease (NCFAD) with 3–4-week-old, weaned, Landrace × Duroc × Large White crossbred piglets obtained from a local supplier in Winnipeg. The use of animals for the experiments was approved by the Animal Care Committee (ACC) at the Canadian Science Center for Human and Animal Health (CSCHAH), under the Animal User Document AUD-C-21-003. All procedures in the experiments were performed in compliance with the Canadian Council for Animal Care and the Canadian Veterinary Medical Association guidelines. Upon arrival, the pigs were randomly assigned to four designated animal cubicles (N2590: pigs #1–10, N2600: Pigs #11–20, N2680: pigs #21–30 and N2650: pigs 31–39 for the ASFV Estonia 2014 study and N550: #41–50, N2520: #51–60, N2510: #61–70 and N2480 #71–80 for the ASFV Malta’78 study) and provided with a commercial ration twice a day with ad libitum water.

### 2.2. Virus Inoculations

Following one week of acclimatization, the pigs in Experiment 1 were infected with ASFV Estonia 2014 [21] and Experiment 2 with ASFV Malta’78 [22] oronasally at a dose of 2 × 10^5^ TCID_50_/pig in 2 mL (1 mL oropharyngeal and 0.5 mL per nostril). The viruses used were propagated in porcine primary leukocytes (PPLs) isolated from heparinized whole blood collected from a healthy pig according to the NCFAD virus isolation and propagation protocol. The infected PPLs were incubated at 37 °C in a 5% CO_2_ incubator for 7 days and monitored for the appearance of hemadsorption (HAD), where they were frozen at −80 °C for at least 4 h and thawed to harvest the cell supernatant. The supernatants were titrated in porcine alveolar macrophage (PAM) cells using the NCFAD standard operating procedures [23]. Briefly, 10-fold dilutions of each virus were inoculated into 90% confluent PAM cells in Minimum Essential Medium (MEM) supplemented with 1% Gentamicin, 1% Glutamax and 2% FBS (all from Thermo Scientific, Burlington, ON, Canada). Following a 72 h incubation at 37 °C in a 5% CO_2_ incubator, plates were fixed and stained with anti-ASFV polyclonal pig serum (ASF-CP, ASF-European Reference Laboratory, Madrid, Spain) and HRP-conjugated goat anti-swine polyclonal antibody (Peroxidase AffiniPure^®^ Goat Anti-Swine IgG, Cat# 114-035-003; Jackson ImmunoResearch, West Grove, PA, USA). Virus titers were calculated based on a 50% tissue culture infectious dose (TCID_50_) assay. Post inoculation of the pigs, the amount of virus in the inoculum was further confirmed by back titration on PAMs as explained above.

### 2.3. Sample Collection

Following infection, four pigs from each group were randomly selected on 1, 2, 3, 4, 5, and 7 days post-infection (dpi), and humanely euthanized. After 7 dpi, pigs showing the most advanced clinical signs were selected for euthanasia. Prior to euthanasia, 2 mL of whole blood in EDTA from each animal was collected. Upon euthanasia, lymphoid tissues, SILN, SMLN, SCLN, PLN, PFLN, and GHLN, spleen, and tonsils were collected in the respective order, starting from the most superficial to internal tissues by performing a complete necropsy. A portion of tissues was fixed in 10% neutral phosphate-buffered formalin, and the rest were stored at −80 °C until tested by real-time PCR as described previously [23].

### 2.4. Real-Time PCR

Tissue homogenates (10% *w*/*v*) were prepared from all tissue samples using Precellys homogenizing kits with the use of the Precellys^®^ Touch tissue homogenizer (Bertin technologies, Rockville, MD, USA) according to the NCFAD standard protocol. Approximately 0.1 g of tissue was weighed using a laboratory scale and transferred to homogenizing tubes containing 1 mL of sterile PBS. The tubes were closed tightly, and tissues were homogenized twice at 3 × 10 s cycles at 5000 RMP. The homogenates were spun down at 2000× *g* for 20 min at 4 °C, and 55 µL of clarified supernatant was used for nucleic acid extraction.

Total nucleic acids from whole blood and tissue homogenates were extracted using Magmax Core Nucleic Acid Purification kit (Life Technologies Corporation, Austin, TX, USA) according to the manufacturer’s instructions with the help of the Magmax ^TM^ Express—96 Deep Well Magnetic Particle Processor (Thermo Fisher Scientific, Waltham, MA, USA). ASF genomic material was detected using the modified Zsak assay quantitative real-time PCR (qRT-PCR) assay described by Wang et al. [24], which targets a highly conserved region of the B646L (p72) open reading frame. Real time PCR reactions were prepared using TaqMan™ Fast Virus 1-Step Master Mix (Thermo Fisher Scientific) and were amplified using the Quant Studio 7 Pro Real Time PCR System instrument (Applied Biosystems, Carlsbad, CA, USA), using the recommended cycling conditions (50 °C for 5 min, 95 °C for 20 s, followed by 95 °C for 3 s and 60 °C for 30 s) for the TaqMan™ Fast Virus 1-Step Master Mix, and run for 40 cycles. The cycle threshold (Ct) values were analyzed by the Design and Analysis 2.5.1 software (Thermofisher Scientific).

### 2.5. Histopathology, Immunohistochemistry (IHC) and In Situ Hybridization (ISH)

To visualize the lesions induced and the distribution of ASFV genetic material and antigens in the SILNs following ASF infection, histopathology, IHC, and ISH were performed. Tissues were fixed in 10% neutral phosphate-buffered formalin, processed, sectioned at 5 µm, and stained with hematoxylin and eosin (HE) for histopathological examination.

For IHC, paraffin-embedded formalin-fixed tissue sections were quenched for 10 min in aqueous 3% hydrogen peroxide. Epitopes were retrieved using an in-house glyca retrieval solution in a Biocare Medical Decloaking Chamber™ (Biocare Medical, Pacheco, CA, USA). Then the tissue sections were incubated with the primary antibody F88ASF55-2-1 specific for ASFV A137R [25] and or MAC387 (Bio-Rad, Mississauga, ON, USA) specific for Macrophages/Monocytes/Granulocytes for thirty minutes and visualized using an anti-mouse horse radish peroxidase (HRP) labeled polymer (Envision^®^ + system, Agilent, Santa Clara, CA, USA) and diaminobenzidine (DAB). The sections were then counterstained with Gill’s hematoxylin (in-house).

For ISH, 5 μm paraffin-embedded formalin-fixed tissue sections were processed according to the user manual for the RNAscope^®^ 2.5 HD Assay—RED kit (Advanced Cell Diagnostics USA, Newark, CA, USA) using the V-AFSV-01 probe (# 813471, Advanced Cell Diagnostics USA). The sections were then counterstained with Gill’s 1 hematoxylin, dried, and cover-slipped.

### 2.6. Graphical Presentation of Data and Statistical Analysis

The graphs were prepared using GraphPad Prism 10 (version 10.4.1) for Windows (www.graphpad.com (accessed on 10 September 2025), GraphPad Software, Boston, MA, USA). Individual CT values were plotted on XY-type graphs. Daily average Ct values were calculated and plotted with standard error of the mean (SEM) bars with dotted connecting lines.

## 3. Results

### 3.1. Experiment 1 (ASFV Estonia 2014)

#### 3.1.1. Clinical Findings of Experiment 1

The pigs in Experiment 1 (ASFV Estonia 2014) developed a mild diarrhea during the acclimatization period. They were treated with Ceftiofur, 100 mg/mL intramuscularly and oral electrolytes, and monitored for 14 days until fully recovered. Then they were infected oronasally with ASFV Estonia 2014 as explained above. Rectal temperatures of the pigs started increasing from 3 days post-infection (dpi), and by 5 dpi, most pigs developed high fever (>41 °C) (Supplementary Table S1), lethargy, loss of appetite, and dry feces. Blood was observed in feces in one pen starting 6 dpi, and a few pigs showed ataxia and labored breathing. Four pigs; #39 on 7 dpi, #9 on 8 dpi, #35 on 10 dpi, and #18 on 11 dpi, were found dead during the study. One pig developed seizures (Pig #8 on 9 dpi) and two developed hypothermia (Pigs #21 and #24 on 9 dpi), so they were euthanized humanely due to reaching end points. The study was concluded at 11 dpi, and the remaining pigs (*n* = 5) were humanely euthanized.

#### 3.1.2. ASFV Genomic Detection Dynamics in Whole Blood, Central Lymphoid Organs, and Peripheral Lymph Nodes

**Figure 1:**
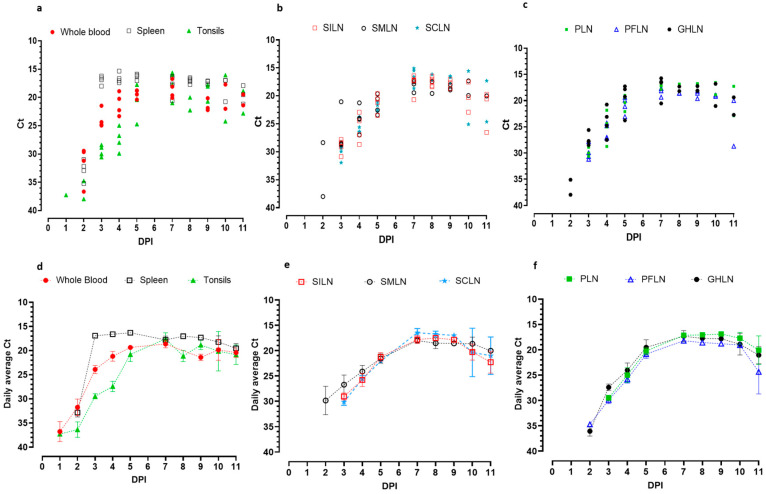
ASFV Estonia 2014 viral genomic distribution in individual pigs (**a**–**c**) and daily average detections (with standard error of mean) in (**d**–**f**) in whole blood, spleen, tonsils, (**a**,**d**) superficial inguinal lymph nodes (SILN), submandibular lymph nodes (SMLN), superficial cervical lymph nodes (SCLN) (**b**,**e**), popliteal lymph nodes (PLN), pre-femoral lymph nodes (PFLN), and gastro-hepatic lymph node (GHLN) (**c**,**f**). The error bars represent the standard error of the mean (SEM) of the Ct values in each time point for a given sample type.

In pigs from Experiment 1, the first clinical sample to indicate the presence of the ASFV genome was the tonsils. On 1 dpi, one out of four sampled pigs had detectable levels (Ct = 37.3) of ASFV genome in their tonsils (Figure 1a). No viremia was detected on 1 dpi.

On 2 dpi, two out of four sampled pigs had detectable levels of ASFV genomic material in their tonsils (Ct 34.7 and 37.9). From 3 dpi onwards, the ASFV genome was detected in the tonsils of all the pigs sampled till the end of the experiment. The highest level of ASFV genome detection in tonsils is observed on dpi 7 (Average Ct 17.57; Figure 1d).

ASFV genome in blood (viremia) was first detected on 2 dpi in all 4 sampled pigs (Ct 29.6, 31.2, 29.4, and 36.6; Figure 1a). Thereafter, all pigs sampled on each sampling day indicated the presence of ASFV genomic material in their blood (Daily average Ct 17.5–23.9). The highest average detection (the lowest average Ct) of ASFV genome in whole blood is observed on 9 dpi (Average Ct 17.59; Figure 1d) in 3/3 sampled pigs. On the last day of sampling (11 dpi), the average Ct was 19.37 (Figure 1d).

Viral genome detection in the spleen started at 2 dpi in all 4 sampled pigs (Ct 30.9, 32.2, 32.9, and 35.9), and continued to be detected in all sampled pigs until the end of the study (Figure 1a). The lowest average Ct value was observed on 8 dpi (Ct 17.02). Daily average ASFV genomic detection in spleen remained < Ct 20 by the end of the study, which was only ~3 Ct values higher than the highest average detection (Figure 1d).

Out of the superficial lymph nodes, the first one to show evidence of ASFV genomic presence was SMLN, in 2/4 sampled pigs (Ct 38.0 and 28.4) on 2 dpi (Figure 1b). From 3 dpi onwards, all sampled SMLNs were positive for ASFV genomic material. Average detection increased with declining Ct, daily showing the lowest Ct value on 7 dpi (Average Ct 17.99; Figure 1e). Thereafter, the Ct values gradually increased, resulting in an average Ct value of 20 by the last sampling point on 11 dpi (Figure 1e).

SILN tested positive for ASFV genome on 3 dpi, with all 4 tested SILNs containing ASF genomic material (Ct 30.8, 29.1, 27.8, and 28.3; Figure 1b). All SILN samples collected thereafter tested positive till the end of the study (11 dpi). On average, dpi 7–9 displayed the highest detection window with the lowest Ct values (Average Ct 17.79–17.82; Figure 1e), displaying 8 dpi as the lowest average Ct value detected date (Average Ct 17.5). After 9 dpi, the Ct values started increasing with apparent variability in detection levels between the SILNs collected towards the end of the study (Figure 1b). On the last day of sampling (11 dpi), the average Ct of ASF genomic material in SILN is 22.27 (Figure 1e).

First detection of ASFV genomic material in SCLN was on 3 dpi in all tested pigs (Ct 29.2, 29.3, 29.9, and 31.9; Figure 1b). Thereafter, the detection levels became stronger with the highest average detections observed between 7 and 9 dpi (Ct 16.46, 16.72, and 16.99; Figure 1e). Following 9 dpi, the Ct values started increasing towards the study’s end on 11 dpi (Average Ct 20.98, Figure 1e).

Detection of the ASFV genome in PFLN also began on 3 dpi in all tested pigs (Ct 31.2, 29.9, 27.3, and 30.7) and continued to be detected in all sampled PFLNs till the end of the study (Figure 1c). It appeared that the highest detection levels were between 7 and 9 dpi (Average Ct 18.21, 18.59, and 18.78), similar to the other superficial lymph nodes, and the Ct values started increasing thereafter (Figure 1f). On day 11, when the study concluded, the average ASF detection Ct in PFLN was 24.35 (Figure 1f). The two pigs sampled on 11 dpi showed Ct values distinctly variable from each other with ~8 Ct difference (Ct 20 and 28.69; Figure 1c).

ASFV genomic detection in popliteal lymph nodes (PLN) was also observed on 3 dpi in all samples (Ct 29.99, 30.62, 28.43, and 28.88), which continued till the end of the study on 11 dpi (Figure 1c). Following the same pattern, the Ct values of ASFV genomic detection are lowest between 7 and 10 dpi (Figure 1c,f). By the time the study ended (11 dpi), average Ct on dpi 11 was 20.08, and the detection level in the last two pigs was variable, with a difference of ~5 Ct values (Ct 17.28 and 20.08; Figure 1c).

In gastrohepatic lymph nodes (GHLN), ASFV genomic material was detected as early as 2 dpi in three out of four sampled pigs (Ct 35.09, 35.11, 35.82; Figure 1c). After 2 dpi, GHLNs remained positive for the ASFV genome in all animals sampled. The lowest average Ct was detected on 7 dpi (Ct 17.31). The Ct values gradually increased thereafter and were observed at an average Ct of 21.04 on the last day of sampling (Figure 1f).

When all sample types were considered, the key trend in the average Ct values of ASFV genome detection across time was similar. The detection started weakly with a relatively higher variability in the early sampling points, then peaked for about 2 days, where the Ct values were uniform towards the peak detection. Following the strongest peak detection, the Ct values showed a declining trend towards the final two sampling points, where the variability between Ct values at a given time point was higher. The spleen showed the lowest variability compared to other sample types, at each time point from the beginning to the end of sampling.

#### 3.1.3. Histopathology and ASFV Detection by Immunohistochemistry (IHC) and In Situ Hybridization (ISH)

**Figure 2:**
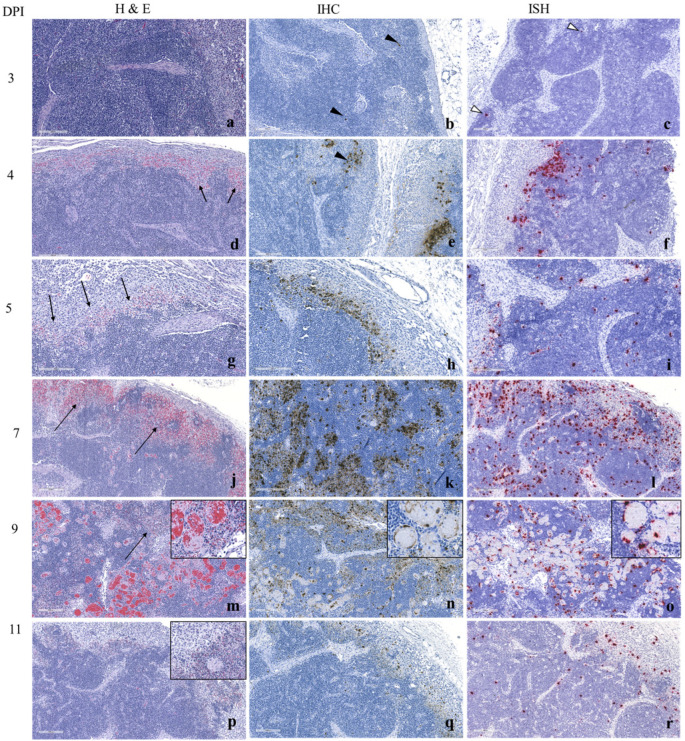
Histopathological observations and the distribution of ASFV in SILNs following oro-nasal inoculation of moderately virulent ASFV Estonia 2014 in piglets. No significant histopathological changes at 3 dpi; (**a**) Scattered single-cell staining for ASFV antigen observed by IHC, (**b**) arrowheads and ASFV RNA detected by ISH show a similar distribution, (**c**) arrowheads. Multifocal areas of hemorrhage in the cortex and cortico-medullary junction at 4 dpi, (**d**) arrows. Prominent patches of scattered single-cell positive staining by IHC (**e**) arrowhead, and detection of viral genomic material by ISH (**f**), show the same distribution and intensity. Multifocal necrosis with hemorrhage primarily along the corticomedullary junction at 5 dpi (**g**) arrows. Viral antigen (**h**) and viral RNA (**i**) were detected corresponding with the necrotic lesion and scattered throughout the tissue in macrophage-like cells. Widespread hemorrhages and necrosis at the corticomedullary junction and multifocally throughout the cortex at dpi 7 (**j**) arrows)) Extensive AFV antigen in the corticomedullary junction and some scattered cells throughout the cortex at 7 dpi (**k**). Presence of viral nucleic acid was decreased comparatively at this time point (**l**). Extensive necrosis and hemorrhage throughout the node at 9 dpi (**m**), arrow)) including degeneration of endothelial cells (**m**, inset shows higher magnification of necrotic area). Viral antigen (**n**) and viral nucleic acid (**o**) were detected multifocally throughout the node in lesser amounts than observed at dpi 7. However, viral antigens were observed within the endothelial cells of blood vessels (**n**,**o**) insets. Moderate necrosis observed at 11 dpi (**p**), but immunostaining greatly decreased (**q**,**r**). Images were taken using a 10× objective (100× magnification), excluding the insets, which were taken using higher magnifications.

In the ASFV Estonia 2014 infected pigs, histopathological changes or ASFV antigen detection, were not observed by H and E staining or immune staining in SILNs until 3 dpi. At 3 dpi, immunostaining of <10 single cells in the medullary sinuses (Figure 2b) is observed. ASFV in situ hybridization signals show a similar distribution (Figure 2c). By 4 dpi, with H and E staining, one of the 4 SILNs showed evidence of hemorrhages in the cortex and cortico-medullary junction (Figure 2d). Immunostaining (Figure 2e) and hybridization signals (Figure 2f) observed in the medulla ranged from scattered single cells to prominent patches. A similar finding was observed at 5 dpi with scattered areas of necrosis along the corticomedullary junction (Figure 2g); however, hybridization signals (Figure 2h) are slightly more intense than the immunostaining (Figure 2i).

Based on morphology, the stained cells appeared to be macrophages and/or dendritic cells. Double immunostaining confirmed the presence of viral antigen in macrophages (Supplementary Figure S1a); however, several infected cells could not be identified as macrophages and were most likely dendritic cells (Supplementary Figure S1b).

At 7 dpi, widespread hemorrhage and necrosis at the corticomedullary junction and multifocally throughout the cortex are observed (Figure 2j), and there is evidence of lymphocytolysis. Extensive positive immunostaining for viral antigen is observed at the corticomedullary junction and within scattered macrophages and/or dendritic cells throughout the cortex (Figure 2k). Despite the degeneration and loss of lymphocytes, viral antigen could not be detected within these cells. There were still abundant hybridization signals detected at 7 dpi (Figure 2l); however, there seems to be less viral nucleic acid detected compared to the viral antigens.

By 9 dpi, extensive necrosis and hemorrhage could be observed throughout the lymph node (Figure 2m), including degeneration of endothelial cells (Figure 2m, inset). The immunostaining pattern was similar to what was observed at 7 dpi; however, viral antigen could be observed extensively within the endothelial cells of blood vessels (Figure 2n). By 11 dpi, moderate necrosis was still evident (Figure 2p); however, the immunostaining was greatly reduced (Figure 2q).

### 3.2. Experiment 2 (ASFV Malta’78)

#### 3.2.1. Clinical Findings of Experiment 2

The pigs in Experiment 2 (ASFV Malta’78) started showing increased rectal temperatures 4 dpi onwards, reaching over 41 °C by 5 dpi (Supplementary Table S2). Pigs were lethargic with poor appetite on 5 dpi, and one pig (#71) showed signs of rectal bleeding. Starting at 7 dpi, blood was noticed on the thermometer after taking the rectal temperature of several pigs, and blood clots were noticed in the feces in some pens. Some pigs also showed hemorrhages on the skin, ataxia, and dyspnea. During the study, one pig was found dead (#77 on 10 dpi), and one pig (#80) was humanely euthanized (on 6 dpi) as they developed rectal bleeding and or hypothermia. The study was concluded on 18 dpi, and the three remaining pigs were humanely euthanized.

#### 3.2.2. ASFV Genome Detection Dynamics in Whole Blood, Central Lymphoid Organs, and Peripheral Lymph Nodes

**Figure 3:**
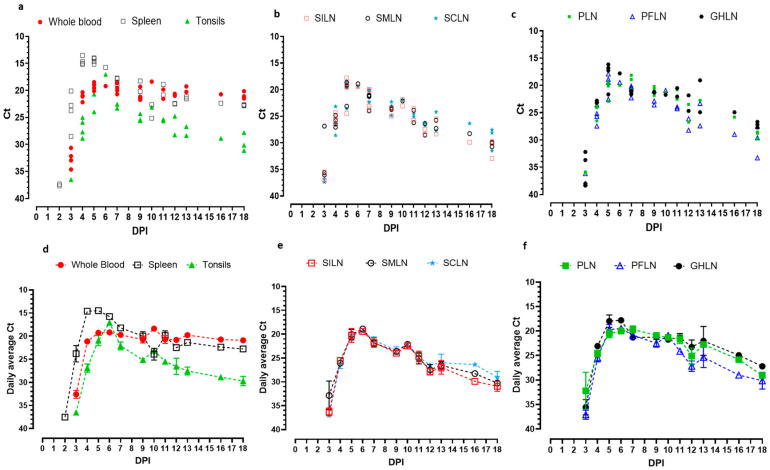
ASFV Malta’78 viral genomic distribution in individual pigs (**a**–**c**) and daily average detections (with standard error of mean Ct) in (**d**,**f**) whole blood, spleen, tonsils, (**a**,**d**) superficial inguinal lymph nodes (SILN), submandibular lymph nodes (SMLN), superficial cervical lymph nodes (SCLN), and (**b**,**e**) popliteal lymph nodes (PLN), pre-femoral lymph nodes (PFLN), and gastrohepatic lymph node (GHLN) (**c**,**f**). The error bars represent the standard error of the mean (SEM) of the Ct values in each time point for a given sample type.

In ASFV Malta’78 infected pigs, the very first detection of the ASFV genome is observed in the spleen samples (Figure 3a). Two out of four (2/4) spleens sampled have low levels of ASFV genome detections (Ct 37.3 and 37.7) at 2 dpi (Figure 3a). Thereafter, the detection levels in spleens increased rapidly and remained consistently strong till the study ended at 18 dpi. The lowest Ct values of ASFV genomic detection in any given tissue are shown in the spleens of this study between 4 and 6 dpi (Average Ct 14.6–15.74; Figure 3d), with one spleen on 4 dpi showing a Ct value as low as 13.51. Starting from 7 dpi, the Ct values continued to increase. By the last day of sampling on 18 dpi, the average Ct value for ASFV genomic detection is 22.74 (Figure 3d).

The earliest viremia is observed on 3 dpi with the detection of ASFV genomic material in all four sampled pigs’ whole blood (Ct 34.6, 32.9, 32.2, and 30.6; Figure 3a). The highest viremia (lowest Ct values) was reached between 5 and 7 dpi, as indicated in Figure 2a,d (Lowest average Ct 19.19 on 6 dpi). ASFV genomic detection in blood remained consistently high throughout the study (Figure 3a). At the end of the study (18 dpi), the average detection of viremia is 20.91 (Figure 3d).

On 3 dpi, the ASFV genome started to be detected in tonsils, all sampled lymph nodes, and lymphoid organs. On 3 dpi, one of four sampled tonsils indicate ASFV genome (Ct 36.5; Figure 3a). The ASFV genome was detected in tonsils in all sampled pigs thereafter, apart from pig #48, in which the tonsil tested negative on 7 dpi. The lowest Ct values for ASFV genomic material in tonsils are identified between 5 and 7 dpi (Average Ct 20.94–22.12), with the lowest average Ct on 6 dpi (Average Ct 17.06; Figure 3d). The Ct values started increasing rapidly from 7 dpi towards the end of the study (Figure 3d), more strikingly compared to ASFV Estonia 2014 infected pigs. On the last day of sampling (18 dpi), the average ASFV genomic detection in tonsils is 29.71 (Figure 3d). The detection levels (Ct values) between samples appeared more variable towards the end, where the final samples depicted quite high and variable Ct values of 31.14, 27.83, and 30.15 (Figure 3a).

ASFV genomic detection in SILNs is observed starting on 3 dpi, when two of four sampled pigs show low levels of detection (Ct 35.5 and 37.0; Figure 3b). Thereafter, ASFV genome detection in SILN rapidly increases, with the highest and the most consistent levels of detection observed between 5 and 7 dpi (average Ct 20.25, 19.4, and 21.72, Figure 3b,e). From 8 dpi on, the Ct values started increasing, and on the last sampling day of 18 dpi, the average detection is Ct 30.93 (Figure 3b,e). On 18 dpi, all three sampled SILNs are positive for ASF, and two of three show Ct >30 (Ct 30.07 and 30.74; Figure 3b).

SMLNs displayed initial detection of ASFV genome on 3 dpi, in three out of four sampled pigs (Ct 35.6, 26.8, and 36.0; Figure 3b). The highest detection levels in SMLN are found between 5 and 7 dpi (Average Ct 20.04–21.85), with the lowest Ct 18.90 on 6 dpi (Figure 3b,e). Thereafter, the Ct values markedly increased gradually on each sampling day, resulting in a final average Ct of 30.93 on 18 dpi (Figure 3e) with two out of three pigs showing Ct values over 30 (Ct 29.7, 30.08, and 32.95; Figure 3b).

ASFV genome detection in SCLN began on 3 dpi, at lower levels in two out of four sampled pigs (Ct 36.75 and 37.36), as shown in Figure 3b. Such as the aforementioned superficial LNs, the highest average ASFV genomic detection in SCLNs is observed between 5 and 7 dpi (Ct 20.1, 19.4, and 21.2; Figure 3e). The Ct values start increasing thereafter, resulting in an average Ct of 29.04 (Figure 3e) on the last day of sampling (18 dpi), where three pigs are sampled, and one of the three positive pigs showed a Ct of 31.48, while the other two pigs show a Ct < 30 (Figure 3b).

PFLN indicates positive ASFV genomic detection in two of four sampled pigs, on 3 dpi (Ct 38.0 and 36.2; Figure 3c). The highest average detection is between 5 and 6 dpi (Ct 19.69–19.47; Figure 3f). Following that, 7 dpi on, Ct values started increasing towards 18 dpi. The average Ct on 18 dpi is 30.14 (Figure 3f), and two out of three pigs show Ct values over 30 (30.07 and 30.74; Figure 3c).

PLN showed lower level initial ASFV genome detection at 3 dpi, in 3/4 sampled pigs (Ct 35.89, 36.0, 32.2). Thereafter, ASFV genomic material is consistently detected in all sampled pigs, and the relatively higher detection levels are observed between 5 and 7 dpi (Average Ct 20.51, 19.99, and 19.63; Figure 3f). Thereafter, the Ct values start to increase gradually. On 18 dpi, the average Ct in PLN is 29.04 (Figure 3f).

All GHLNs that were sampled on 3 dpi were positive for ASF genomic material (Ct 32.2, 33.68, 38.0, and 38.36). Thereafter, high levels of ASFV genomic material are detected in GHLN (Figure 3c). The highest detection window is observed between 5 and 6 dpi (Ct 17.98 and 17.8; Figure 3c,f), and after 11 dpi, the Ct values start to increase, with increasing variability in detection levels between samples (Figure 3c). On 18 dpi, the average Ct of the ASF genome in GHLN is 27.20 (Figure 3f).

The trend kinetics of ASFV genome detection in the ASFV Malta’78 experiment were similar. At the early detection points, the Ct values appeared at weak levels with more variability, and when the detections peaked, variability was lowest with uniform Ct values. Whole blood and spleen peak detections coincided, whereas in the tonsils, the peak detection point happened one day later. In the superficial lymph nodes and GHLN, the trend was uniform with coinciding peak detection points. Following the peak detection, the average Ct values indicated a gradual decline over the course of the next multiple sampling points. The tonsils showed a trend of rapid decline in Ct values, indicating faster clearance of viral genome compared to blood and spleen. The trend of viral genome detection declining in SILN, SMLN, and SCLN appeared similar and almost superimposed 7 dpi onwards. PLN and GHLN seemed to follow the same trend of clearance, whereas PFLN showed a slightly higher decline from 13 dpi. On the final sampling day of 18 dpi, more variability in Ct values was observed in the SILN, SCLN, and PFLN compared to the other samples.

#### 3.2.3. Histopathology and ASFV Detection by Immunohistochemistry (IHC) and In Situ Hybridization (ISH)

**Figure 4:**
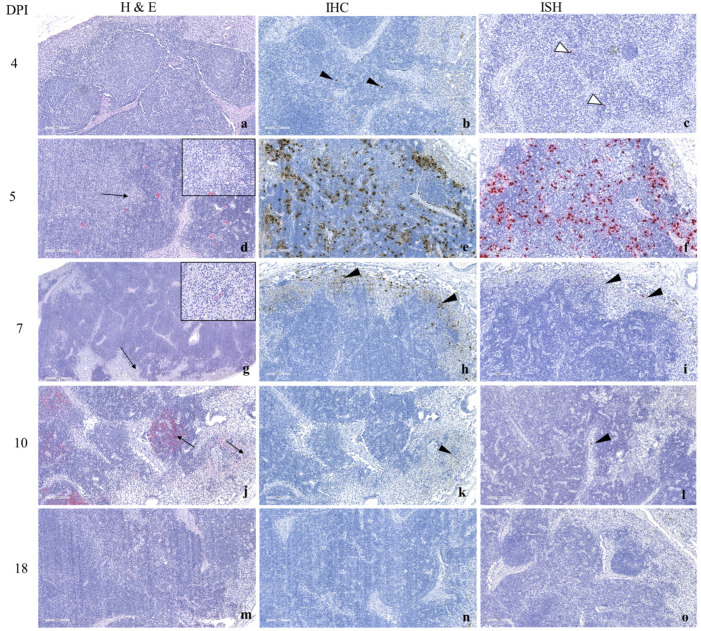
Histopathological observations and the distribution of ASFV in SILNs following oro-nasal inoculation of moderately virulent ASF Malta’78 in piglets. At 4 dpi, no lesions were observed in H and E sections (**a**). Scattered individual cells with morphological appearance of macrophages were observed by IHC (**b**) arrowheads and ISH (**c**) arrowheads. By 5 dpi, a few small necrotic areas were observed in the medulla (**d**) arrow; inset shows higher magnification of the necrotic areas. Abundant ASFV antigen (**e**) and ASF viral RNA (**f**) were detected in cells morphologically consistent with macrophages and dendritic cells. By 7 dpi, areas of medullary necrosis were present (**g**) arrow; inset shows higher magnification of the necrotic area. A lesser amount of ASFV antigen (**h**) and viral RNA (**i**) was detected compared to 5 dpi. At dpi 10, hemorrhage and necrosis primarily at the corticomedullary border (**j**) were observed with associated weak immunostaining (**k**,**l**). By 18 dpi, reactive hyperplasia was seen in the SILN tissue (**m**), and no visible staining was observed by IHC (**n**) or ISH (**o**). Images a–m were taken using a 10× objective (100× magnification), excluding insets, which are higher magnification. Images (**a**–**m**) taken using a 10× objective (100× magnification), excluding insets, which are higher magnification.

In ASFV Malta’78 infected pigs, similar but delayed and less severe lesions were observed in SILNs compared to those in ASFV Estonia 2014 infected pigs. ASFV antigens and viral RNA (Figure 4b,c) are first detected in SILN on 4 dpi, although no microscopic lesions are observed. At 5 dpi, small areas of necrosis are visible in the medulla (Figure 4d; inset shows higher magnification of necrotic area), and a moderate amount of ASFV antigens is detected by IHC (Figure 4e), and a moderate to large amount of viral RNA is detected by ISH (Figure 4f). The stained cells showed the morphological appearance of macrophages and dendritic cells. By 7 dpi, scattered areas of necrosis in the medulla are still observed (Figure 4g) but are only discernible at higher magnification (Figure 4g, inset). A distinctly lesser amount of viral antigen was detected in macrophages and dendritic cells compared to the previous time point (Figure 4h). Compared to the amount of viral antigen detected, there was significantly less viral RNA present (Figure 4i). From 10 dpi onwards, hemorrhage and necrosis, especially at the corticomedullary border, are observed (Figure 4j), and weak to mild IHC staining is observed (Figure 4k). By 18 dpi, the SILN tissue showed reactive hyperplasia on the H and E sections (Figure 4l). No visible staining on IHC can be observed by 18 dpi (Figure 4m).

### 3.3. End-Point Detection of ASFV Genome in Spleen and SILN of Dead and Euthanized Pigs

A total of nine pigs succumbed to ASFV infection during the two experiments. The Ct values of ASFV genomic detection in spleen in comparison to that of the SILN of these pigs are indicated in Table 1.

## 4. Discussion

Previously, we reported the suitability of SILNs as a reliable and convenient sample type for screening dead pigs for ASF [20]. In that study, the conclusions were made based on the results from SILNs, the last/final whole blood and spleen samples collected from found-dead or euthanized pigs that reached humane endpoint following experimental inoculation with highly or moderately virulent ASFV strains. While we found comparable levels of ASFV genome in SILNs and spleens collected in that study, Pikalo et al., using moderately virulent ASFV Estonia 2014, reported higher variability and low levels of genome in the SILNs collected during the early stages of infection (4 dpi) [19]. The assumptions were that the virus was not yet distributed to peripheral lymphoid organs at the time of sampling and/or accidental inclusion of fatty or connective tissues when the SILNs were harvested from the pigs. This raised questions regarding the suitability of SILN for ASFV detection in pigs during early stages and those that survive moderately virulent ASFV infections.

The present study was designed to answer those questions by exploring the kinetics and chronological distribution of the ASF viral genome in superficial lymph nodes, particularly in SILNs, in relation to the viremia following ASFV infection via the natural (oronasal) route. For the study, two moderately virulent ASFV strains, ASFV Estonia 2014 and ASFV Malta’78, were used. ASFV Estonia 2014 has shown variable mortality in pigs [26]. In our study, all pigs that received ASFV Estonia 2014 developed high fever, lethargy, and ataxia; four pigs were found dead, and one was euthanized as it developed seizures (Table 1). The study was terminated on 11 dpi. Pigs inoculated with ASFV Malta’78 developed less severe transient clinical signs; only one pig was found dead, and one was humanely euthanized due to bleeding from the rectum and hypothermia.

During ASFV oronasal infection, the virus enters via the tonsils or dorsal pharyngeal mucosa, and then spreads to the mandibular or retropharyngeal lymph nodes, from where it spreads by viremia to other lymphoid organs such as spleen, bronchial, gastro hepatic, and mesenteric lymph nodes [27,28]. In our study, the earliest onset of viremia was detected on 2 dpi in Experiment #1 with ASFV Estonia 2014 infection, and on 3 dpi in Experiment #2 with ASFV Malta’78 infection (Table 2). The difference in the onset of viremia observed could be due to the subtle difference in the virulence of the two viruses. This difference was also noticed in the ASFV genome detection kinetics in the tonsils, the primary replication site. In Experiment #1, one out of four ASFV Estonia 2014 infected pigs showed the presence of ASFV genomic material in the tonsils as early as 1 dpi, followed by detection in SMLN on 2 dpi. In contrast, in ASFV Malta’78 infected pigs, the presence of the ASFV genome in tonsils and SMLN was detected from 3 dpi. Interestingly, in the ASFV Malta’78 infected pigs, a low level of the ASFV genome was detected in the spleen on 2 dpi.

In both experiments, the ASFV genome was detected in peripheral lymphoid tissues, including SILN, within 3 dpi, and the amounts were comparable to those in the whole blood. Thereafter, the viral load increased rapidly in the blood as well as in the lymphoid tissues. In ASFV Estonia 2014, infected pigs, the ASFV genome detection peaked around 4–5 dpi in whole blood, spleen, and tonsils, but only around 7 dpi in peripheral lymph nodes. By 7 dpi, the amount of viral genome detected in peripheral lymph nodes was comparable to that detected in the spleen samples. The ASFV genome remained high in all tissues, including blood, until 9 dpi and started to decrease around 10 dpi. In ASFV Malta’78 infected animals, viral genome peaked around 3–5 days in all lymphoid tissues, and starting 6–7 dpi, the amount of viral genome started to decrease in all lymphoid tissues, including the spleen. In both ASFV Estonia 2014 and ASFV Malta’78 experiments, during the early and late stages of the infection, in addition to the lower levels, high variability of ASFV genome levels in peripheral lymphoid tissues was observed.

In both studies, the histopathological data corroborated the PCR data obtained for the SILNs. Coinciding with the PCR detection, ASFV-infected macrophage-like cells were detected in SILNS by 3 dpi in both experiments, and the numbers gradually increased over time, causing widespread hemorrhages, necrosis, and degeneration of lymphocytes in the SILNs. No viral antigens were detected in affected lymphocytes, suggesting apoptosis due to pro-inflammatory cytokines secreted by the infected macrophages. In ASFV Estonia 2014, infected pigs, by 9 dpi, showed severe degeneration of endothelial cells containing ASFV antigens. Coinciding with increasing Ct values, ASFV antigen expression, and pathological lesions observed in SILN decreased toward the end of both studies, indicating potential signs of viral clearance. The changes were clearly evident in the ASFV Malta’78 study, which lasted up to 18 dpi. While a trend of viral clearance from lymphoid tissues was observed in pigs that survived ASFV Estonia 2014 and Malta’78 challenge in the present study, all pigs that succumbed to ASF during the experiment had comparable levels of ASFV genomic material in the spleen and SILNs (Table 1). The death/euthanasia of those pigs was observed between 6 and 11 dpi, and this finding further strengthened the proposed use of SILN for dead pig surveillance.

The distribution kinetics of the ASFV genome following experimental infection with highly virulent ASFV strains have been explored by others. One study, evaluated the relative chronological expression and distribution of ASFV p30 and p72 genes following an oral inoculation [29]. This study showed detection of p30 and p72 genes in lymph nodes as early as 3 dpi, but lower than that was observed in spleen samples. The p30 and p72 gene levels increased over time and reached comparable levels to those in spleen samples by 9 dpi in pigs that succumbed to the infection [29]. The ASF-associated microscopic lesions in the spleen and lymph nodes aligned with increased ASFV p30 and p72 gene expression kinetics. Unfortunately, the authors did not specify which lymph nodes were used in the study. Another study elucidating the in vivo ASFV transcriptome kinetics following intravenous inoculation of highly virulent ASFV genotype II strain VNUA/HY/Vietnam, detected relatively lower viral loads in the lymph nodes (submandibular, mesenteric and inguinal) compared to spleen, liver and lungs at early stages of the infection (1, 2 and 3 dpi), however reached comparable levels in all organ tissues by 5 dpi [30]. Both these studies show that at early stages of ASF infection, even with highly virulent strains, lower levels of ASFV genome are present in lymph nodes compared to those in the spleen tissue. However, they increase and reach comparable levels in lymph nodes and spleen before the pigs succumbed to the infection, supporting our findings that the SILN are a suitable sample for screening dead pigs.

Highly attenuated ASFV strains, including ASF vaccine candidates, cause no to mild clinical signs and no mortality [31,32,33,34]. Therefore, SILN will not be useful in detecting pigs infected/vaccinated with those strains. Pigs that survive infection with low and moderately virulent ASFV strains could remain viremic for several months and develop long-lasting antibody responses [35,36] with or without signs of disease. Therefore, unless an animal is found dead, whole blood and serum remain the best sample types for detecting ASF.

## 5. Conclusions

Enhanced passive surveillance is needed to maximize early detection of an ASF incursion. Collection of WOAH-recommended sample matrices from dead pigs is time-consuming, requires highly skilled staff, instrumentation, and additional resources for cleaning and disinfecting the contaminated areas after sample collection. Previously, we have shown that SILNs are a convenient and effective sample type for ASFV genome detection in pigs succumbed to highly or moderately virulent ASFV. In this study, we explored the kinetics of ASFV distribution in SILNS following oronasal infection with two moderately virulent ASFV strains and showed that ASFV reaches SILNs by 3 dpi and replicates to the highest levels around 5–7 dpi. The nine pigs that succumbed to ASF had comparable levels of ASFV genomic material in SILN and spleen, confirming that the SILNs are suitable for ASFV detection in dead pigs. The data also showed that the pigs that are likely to survive the infection tend to clear ASFV from the peripheral lymphoid organs. During the early infection and recovery stage, ASFV genome levels could be lower and variable than those in whole blood and spleen. Therefore, SILNs are suitable only for screening pigs that are found dead or euthanized due to severe clinical signs.

## Figures and Tables

**Table 1:** ASFV genomic detection in spleen and SILN of pigs that were found dead or humanely euthanized due to reaching endpoints, during Experiments 1 and 2.

Experiment	ASFV Strain	Found Dead/ Euthanized	Pig Number	DPI at Mortality	Ct in Spleen	Ct in SILN
1	ASFV Estonia 2014	Found dead	38	7	17.63	17.20
1		Found dead	9	8	16.53	16.49
1		Euthanized	8	9	17.61	17.76
1		Euthanized	21	9	17.09	18.73
1		Euthanized	24	9	17.18	17.08
1		Found dead	35	10	16.98	20.31
1		Found dead	18	11	21.17	19.73
2	ASFV Malta’78	Euthanized	80	6	15.74	19.41
2		Found dead	77	10	22.65	23.06

DPI = days post inoculation.

**Table 2:** The earliest detection points and average detection levels (Average Ct) of the ASFV viral genome in superficial lymph nodes compared to whole blood, spleen, tonsils, and GHLN.

	Whole Blood	Spleen	Tonsil	SILN	SMLN	SCLN	PFLN	PLN	GHLN
ASFV Estonia 2014									
First detection point (DPI) (number of positive pigs)	2 (4/4)	2 (4/4)	1 (1/4)	3 (4/4)	2 (2/4)	3 (4/4)	3 (4/4)	3 (4/4)	2 (3/4)
Average Ct at first detection	31.7	32.8	37.3	29.0	33.2	30.1	29.8	29.4	35.3
ASFV Malta’78									
First detection DPI (number of positive pigs)	3 (4/4)	2 (2/4)	3 (1/4)	3 (2/4)	3 (3/4)	3 (2/4)	3 (2/4)	3 (3/4)	3 (4/4)
Average Ct at first detection	32.6	37.5	36.5	36.2	32.8	37.0	37.0	35.9	35.5

## Data Availability

All data related to this study will be made available upon request.

## Supplementary Materials

The following supporting information can be downloaded at: https://www.mdpi.com/article/10.3390/v17111472/s1, Figure S1: Immunohistochemical detection of ASFV antigen in SILN, 5 days following ASFV Estonia 2014 oronasal inoculation. (a) Double immunostaining confirmed the presence of viral antigen (magenta) in macrophages (brown) (b) Detection of viral antigens (magenta)likely in dendritic cells; Table S1: Daily rectal temperatures of the piglets in experiment 1 inoculated with ASFV Estonia 2014; Table S2: Daily rectal temperatures of the piglets in experiment 1 inoculated with ASFV Malta ‘78.
