# Supplementary material for: Kinetics of Viral Genome Distribution in Swine Peripheral Lymphoid Organs Following Oronasal Infection with Attenuated African swine fever virus strains
Source: Viruses. 2025 Nov 4;17(11):1472. doi: 10.3390/v17111472 (PMC12656765; doi:10.3390/v17111472)
Supplement: Supplementary file 1 [file viruses-17-01472-s001.zip › viruses-3915602-supplementary.pdf]

a b

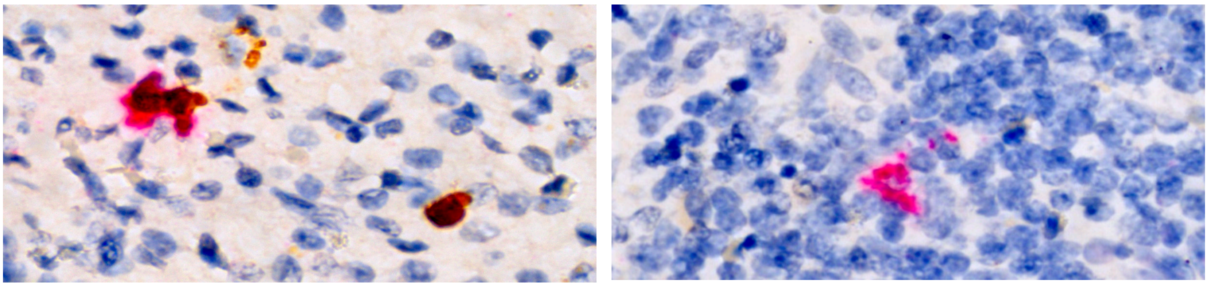

Supplementary Figure S1: Immunohistochemical detection of ASFV antigen in SILN, 5 days following ASFV Estonia 2014 oronasal inoculation. (a) Double immunostaining confirmed the presence of viral antigen (magenta) in macrophages (brown) (b) Detection of viral antigens (magenta)likely in dendritic cells.

Supplementary Table S1: Daily rectal temperatures of the piglets in experiment 1 inoculated with ASFV Estonia 2014

ASFV Estonia 2014 infected piglets											
	Daily Rectal Temperature (C^0)										
Pig #	DPI1	DPI2	DPI3	DPI4	DPI5	DPI6	DPI7	DPI8	DPI9	DPI10	DPI11
1	40	39.8	40								
2	39.7	39.8	39.6	41.1							
3	39.9	39.6	39.7	39.9	41.7						
4	39.9	39.4	39.7	39.9	41.4	41.9	41.3	41.5	41.4	41.2	41.5
5	39.7										
6	39.4	39.3									
7	39.8	39.1	39.4	39.9	41.5	42.4	41.9	41.7	41.7	38.5	
8	40	40	39.9	40.9	41.5	42.1	41.4	42.6	42.6		
9	39.5	39.4	39.6	40.7	40.9	41.9	41.2				
10	39.6	39.9	39.6	40.3	41.8	41.6	40.9				
11	40.3										
12	40.1	40.4	40.6	40.5	41.8	42.2	41.3	41.6	41.4	41.9	41.9
13	40.5	41	40.4	40.7	42.2						
14	40.2	40.1	41.8								
15	40.1	40.6	41.1	41.6							
16	40.3	40.5	40.1	41.1	41.5	41.6	39.4				
17	40.5	40.4	40.9	41.3	42	42.3	42.3	39.2			
18	40.2	40.2	40.1	40.8	41.4	41.4	41.7	41.5	41.5	40.4	
19	40.2	40.4	40.1	40.2	41.5	41.5	41.7	40.8			
20	40	40.2									
21	40.4	39.6	39.8	40	41.5	42.2	42.1	41.7	41.4		
22	39.5										
23	40	39.5	39.8	39.5	41.3	41.6	41.6	41.8			
24	40	39.6	39.3	39.9	41.4	41.9	41.6	41.8	40.6		
25	40	40	40.1	40.2	41.4	41.6	41.9	42.7			
26	40.2	39.7									
27	40.2	39.7	39.8	40.8							
28	40.3	39.9	40	39.8	41	41.9	41.6				
29	40.1	39.9	39.8	40.7	41.5						
30	40.1	39.7	41								
31	39.1	38.9	39.7	39.6	41	41.5	41.6				
32	39.5	39.3	40.5	40.7	41.9	41.8	41.2	41.8	40.8	40.2	
33	39.2	39.5									
34	39.3	39.7	40.2	41.3							
35	39.2	39.5	39.5	40.9	41.9	41.9	42.2	41.9	42.4		
36	39.6	39.1	39	40.7	41.6						
37	39.6										
38	39.4	38.9	39.6	40.8	41.4	41.4					
39	39.5	39.4	40.1								

Supplementary Table S2: Daily rectal temperatures of the piglets in experiment 1 inoculated with ASFV Malta '78

	ASFV Malta '78 infected piglets																	
	Daily Rectal Temperatures (C^0)																	
**Pig number**	**DPI 1**	**DPI 2**	**DPI 3**	**DPI 4**	**DPI 5**	**DPI 6**	**DPI 7**	**DPI 8**	**DPI 9**	**DPI 10**	**DPI 11**	**DPI 12**	**DPI 13**	**DPI 14**	**DPI 15**	**DPI 16**	**DPI 17**	**DPI 18**
**41**	40																	
**42**	39.8	39.7	40															
**43**	39.8	39.7																
**44**	39.8	39.7	39.8	41.6														
**45**	40.2	39.7	40.2	40.9	41.3	41.7	41.5	41	41.2	41.1	41	40.7	40.9					
**46**	39.5	39.7	41	40.7	42													
**47**	39.5	39.8	40	41.6	41.2	41.5	41.9	41.2	41	41.1	40.2	41.2						
**48**	40.3	39.8	40.3	41	41.6	42.1	42.1											
**49**	40.2	39.8	40.6	41.7	41.5	41.4	42.1	41.4	41.5	41.2	40.8	40.5						
**50**	39.3	39.4	39.9	41.7	41.3	41.2	41.6	40.9	40.2									
**51**	39.8	39.5	40.1	41.5	41	41.2	41.1	40.2	40.5	40.1	39.6	39.8	40.3	39.5	40.1	39.5	39.3	39.4
**52**	39.8	39.5	38.9															
**53**	39.7	39.6	39.5	41.6	41.5													
**54**	39.5	39.6	40.3	40.7	41.5	40.9	40.6											
**55**	39.4	39.2																
**56**	39.8	39.7	40.1	40.5	41.2	40.9	41.5	41.1	40.9	40.9	40.9	40.1	40.5	40.3	39.8	39.7	38.9	39.5
**57**	39.5	39.3	39.7	41.1	41.4	41	41.4	40.6	40.6	40.5	40.8	40.9+M21	40.2	40.4	40.5	41.2		
**58**	39.6	39.4	40	41.2	41.4	41.2	41.3	40	40									
**59**	39.9	39.6	40.3	42.1														
**60**	39.8																	
**61**	39.8	39.9	40.4	41														
**62**	39.7	39.5	39.4	39.7	39.9	40.9	41.7	41.2	41.5	41.3	40.4							
**63**	39.5	39.7	40.5	40.1	41.2	41.2	41.3	41.6	41	41.6	41.8	40.4	41					
**64**	39.5																	
**65**	39.6	39.3	38.6															
**66**	39.4	39.5	39.5	40.8	40.7	41.5	41.6	40.7	39.9	40.8	41	40.9	40.5	39.7	39.7	39.2	38.6	39.1
**67**	39.9	39.5	39.7	40.3	41.8	41.5	41.5	41	40.8	41.1	40.2							
**68**	38.8	39.6	39.9	40.7	41.2	41.3	41.6											
**69**	40.7	39.9																
**70**	40	39.8	39.9	41	41.2	41.5	41.8											
**71**	39.7	39.5	39.7	40.7	41.1													
**72**	39.5	39.6	39.2	39.7	41.2													
**73**	39.4	39.7																
**74**	39.9																	
**75**	40	39.6	39.9	40.7	41.5	41.3	41	40.8	40.3	40.2								
**76**	40	39.1	40	41.3														
**77**	39.6	39.3	40	41.1	41.2	41.5	41.7	41.1	40.6									
**78**	39.9	39.8	39.9															
**79**	39.4	39.4	39.6	40.1	41.9	41.3	40.9	40.8	40.1									
**80**	39.7	39.6	40	40.3	42	40												
